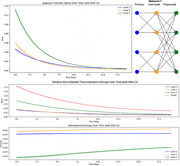# Selective Entropic Vulnerability in a Dynamic Heirarchical Neural Network: Implications for Age‐Related Neurodegeneration

**DOI:** 10.1002/alz70855_103320

**Published:** 2025-12-23

**Authors:** Peter S. Pressman, Peter Foltz

**Affiliations:** ^1^ Layton Healthy Aging and Alzheimer's Disease Research Center, Portland, OR, USA; ^2^ Institute of Cognitive Science, University of Colorado, Boulder, Boulder, CO, USA

## Abstract

**Background:**

The association of Alzheimer's risk with patient age suggests a fundamental relationship with entropy. Artificial neural networks learn by changing probabilistic representations called weights, which in brains are the synaptic‐mediated odds of one neuron communicating with another. Changing these synaptic weights takes physical work (W) by the cell, which directly relates to thermodynamic entropy (S) and temperature (T) by the formula dS/dt = (dW/dt)/T. Using a well‐established model of organic neural networks, we apply this relationship to predict selective vulnerability in the brain to age‐associated neurodegeneration.

**Methods:**

We developed two feedforward hierarchical networks with parallel processing pathways at each level to examine the emergent properties of multi‐level information processing. The base layer contained paired input nodes processing randomized inputs (0.4‐0.6 ±0.05), feeding into expanded middle‐layer and top‐layer processing units, representing primary sensory, unimodal, and heteromodal cortices respectively. Model 1 was columnar, and Model 2 was a convergent pyramidal network. For each iteration, we measured the “work” proxies required for weight adjustments, Lyapunov stability, and approximate thermodynamic and informational entropies for a node at each hierarchical level over 2000 iterations.

**Results:**

In Model 1, the topmost “heteromodal” Layer 3 demonstrated the most significant and variable dynamics. In Lyapunov dynamics analysis, in each cycle Layer 3 starts with the highest variability (0.13 ± 0.08) and ends with stability similar to the other layers (0.00 ± 0.00), indicating substantial stability shifts. For work‐related thermodynamic entropy, in each cycle Layer 3 begins with the highest disorder (0.95 ± 0.18) and shows the largest reduction in entropy over time (AUC: 7.44 ± 1.11). In the informational entropy analysis, Layer 3 starts with lower entropy than the other layers (0.44 ± 0.10) but increases over time to converge on the mean probability of the system input (0.50 ± 0.04). This pattern held for Model 2 as well.

**Conclusion:**

The model predicts that heteromodal cortex undergoes more thermodynamic entropy than primary sensory or unimodal cortex over time, suggesting selective vulnerability to age‐related degeneration due to accumulated structural disorder from information processing demands.